# Glutamine-dependent biosynthetic pathways fuel autoreactive T and B cells in Foxp3 deficiency–mediated disease

**DOI:** 10.1172/jci.insight.200076

**Published:** 2026-09-08

**Authors:** Mohammad Adeel Zafar, Charlotte N. Hill Machado, Jyotirmaya Behera, Yuelin Zhong, Xiao Li, Shakchhi Joshi, Yassine El Fazaa, Virginia Camacho, Peter Georgiev, Kiran Kurmi, Marcia Haigis, Louis-Marie Charbonnier

**Affiliations:** 1 Division of Immunology, Boston Children’s Hospital, Boston, Massachusetts, USA.; 2 Department of Pediatrics and; 3 Department of Cell Biology, Blavatnik Institute, Harvard Medical School, Boston, Massachusetts, USA.; 4 Division of Surgery, Boston Children’s Hospital, Boston, Massachusetts, USA.; 5 Department of Immunology, Blavatnik Institute, Harvard Medical School, Boston, Massachusetts, USA.; 6 Department of Molecular & Cellular Pharmacology, University of Miami Miller School of Medicine, Sylvester Comprehensive Cancer Center, Miami, Florida, USA.; 7 Gene Lay Institute of Immunology and Inflammation of Brigham and Women’s Hospital, Massachusetts General Hospital and Harvard Medical School, Boston, Massachusetts, USA.

**Keywords:** Autoimmunity, Inflammation, Metabolism, Autoimmune diseases, Mouse models, Therapeutics

## Abstract

Foxp3 deficiency causes a profound loss of immune tolerance, unleashing autoreactive T and B cells, lymphoproliferation, cytokine-driven inflammation, and autoantibody production. This autoimmune pathology is fueled by increased glutamine usage, but it remains unresolved whether glutamine is necessary to produce energy or for intermediate metabolite biosynthesis responsible for immunomodulation. Here, we demonstrate that glutamine utilization for biosynthetic pathways supported autoimmune inflammation in the settings of Foxp3 deficiency and dextran sodium sulfate–induced colitis. By employing a model of autoimmunity driven by Treg-specific loss of Foxp3, we showed that this effect is independent of pathogenic Foxp3-deficient Treg reprogramming. Mechanistically, glutamine biosynthetic pathways sustained conventional T cell activation and proinflammatory cytokine production by preventing inosine accumulation and signaling, thus implicating adenosine pathway modulation in autoreactive T cell dysregulation. Conversely, autoreactive B cell activation and autoantibody production relied on glutamine-dependent asparagine availability, which we identified as a targetable vulnerability for autoantibody formation. These findings highlighted glutamine-driven biosynthetic processes as critical drivers of autoimmunity and revealed distinct metabolic vulnerabilities in autoreactive T and B cells that could be targeted for therapeutic intervention.

## Abstract 2

Blocking glutamine metabolism can treat autoimmune diseases by stopping the building blocks that fuel overactive T and B cells, preventing harmful inflammation and tissue damage.

## Graphical abstract

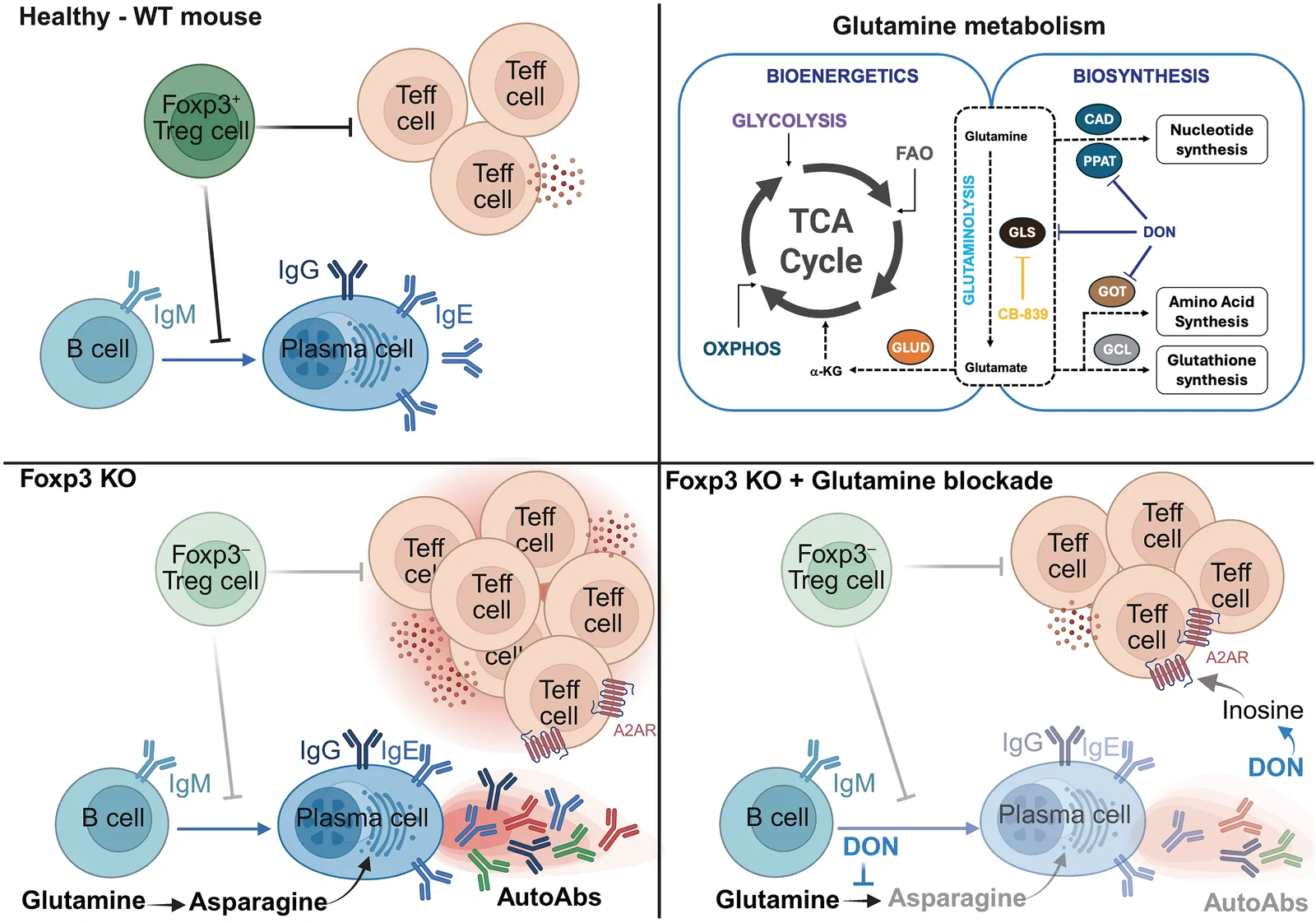

## Introduction

Foxp3^+^ Tregs play a critical role in enforcing immune tolerance to self-antigens and benign antigens (1–3). Loss-of-function mutations in *FOXP3* in humans and its ortholog in mice result in a severe autoimmune lymphoproliferative disease, highlighting the crucial role of Foxp3 in Treg biology (4–7). However, Foxp3 deficiency does not impair Treg development (8–10). While Foxp3-deficient Tregs (ΔTregs) maintain a residual Treg signature, they also acquire effector T cell programs (6, 8, 9). The severity of Foxp3 deficiency–mediated disease in mice can be reduced by CD4^+^ or CD8^+^ T cell depletion, T-bet deficiency, mature B cell deficiency, or Myd88 deficiency, implicating the contribution of innate immune cell maturation and activation and autoreactive T and B cells to disease pathogenesis (11–16).

The activation, differentiation, and effector function of immune cells critically depend on their ability to adapt to and utilize different cellular metabolic pathways owing to their high energy demand. T and B cell activation requires metabolic reprogramming to meet the increased biosynthetic, bioenergetic, and signaling demands (17–20). Current autoimmune therapies largely focus on targeting inflammation or broadly inducing immunosuppression, rather than addressing the underlying cause of disease (21–23). The activity of metabolic pathways is elevated in autoimmune diseases, and metabolic changes are increasingly recognized as important pathogenic processes underlying immune dysregulation. Therefore, metabolically targeted therapies may represent a conceptually novel strategy for treating autoimmune diseases (24). Key metabolic processes such as glycolysis, oxidative phosphorylation (OXPHOS), and nucleotide and amino acid (AA) metabolism are vital for the immune cell survival and function. During inflammation, immune cells undergo substantial metabolic remodeling to meet the bioenergetic requirement for rapid proliferation and effector molecule production (e.g., cytokines, antibody production, etc.). Several reports have highlighted a role for increased glutamine usage in immune cell activation in autoimmune diseases (25–30). In a previous study, we showed that ΔTregs are highly proliferative and exhibit a conventional T cell–like (Tconv-like) metabolic profile associated with a heightened glycolytic rate and high OXPHOS. In contrast to Foxp3-sufficient Tregs, in which OXPHOS is primarily dependent on fatty acid oxidation, OXPHOS in ΔTreg predominantly relies on glucose and glutamine to fuel mitochondrial energy production (9). These results suggest that alterations in glutamine metabolism may be involved in the perpetuation of inflammation driven by autoreactive T and B cells during autoimmunity.

Glutamine, an abundant AA in the serum, serves as an essential fuel source for activated immune cells. Glutamine metabolism plays crucial roles in both bioenergetic and biosynthetic processes, particularly in rapidly dividing cells (31). Glutaminolysis fuels cellular bioenergetics by converting glutamine into glutamate by the enzyme glutaminase (GLS), which is subsequently converted to α-ketoglutarate (αKG). αKG then enters the TCA cycle, providing energy for cellular processes (32). Additionally, glutamine provides substrate for AA biogenesis and glutathione, which is required for protein synthesis and proliferation with the help of glutamic-oxaloacetic transaminase or glutamate cysteine ligase, respectively (33, 34). Glutamine is required for de novo synthesis of purines and pyrimidines, which results from the activity of several enzymes, including phosphoribosyl pyrophosphate amido transferase (PPAT) and carbamoyl-phosphate synthetase, aspartate transcarbamoylase, and dihydroorotase (CAD). Finally, glutamine also generates signaling molecules involved in the activation of immune cells (35).

Here, we show that glutamine-dependent biosynthetic pathways drive autoreactive T and B cell responses in Foxp3 deficiency–mediated disease. Surprisingly, systemic blockade of glutamine usage mitigates autoimmunity in a mouse model of Foxp3 deficiency not in a bioenergetic manner, but by altering glutamine-dependent biosynthetic pathways such as purine metabolites (inosine) and asparagine availability. Pharmacological inhibition of inosine signaling by A2A receptor blockade and supplementation of asparagine specifically reverses the effect of glutamine blockade on T cells and B cells, respectively. Our work provides the basis for development of a potentially novel therapeutic approach to treat autoimmune and inflammatory diseases by targeting glutamine-dependent biosynthetic pathways.

## Results

### Glutamine-dependent biosynthetic pathways contribute to Foxp3 deficiency–mediated disease.

**Figure 1:**
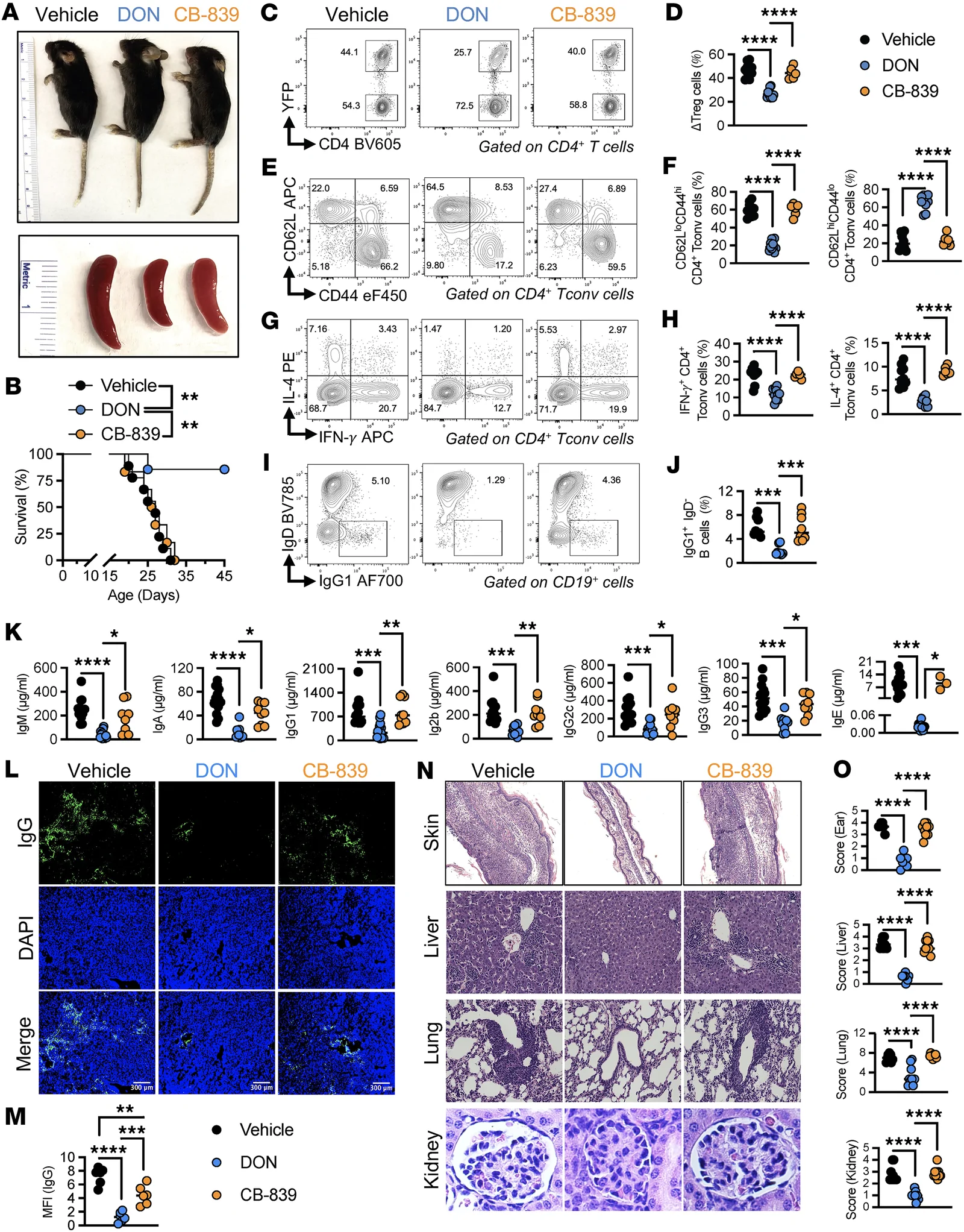
Glutamine-dependent biosynthetic pathways contribute to Foxp3 deficiency–mediated disease. (**A** and **B**) Gross appearance and the respective spleens of 21-day-old *Foxp3*^ΔEGFPiCre^*R26*^YFP^ mice treated with vehicle, 6-diazo-5-oxo-norleucine (DON) or CB-839 (**A**) and survival over time (**B**). The results represent 1 of the 4 experiments. (**C**–**J**) Representative flow cytometric analysis and frequencies (scatterplots and means) of CD4^+^YFP^+^ ΔTregs (**C** and **D**); CD62L^lo^CD44^hi^, CD62L^hi^CD44^lo^ CD4^+^ Tconv (YFP^–^) cells (**E** and **F**); and IFN-γ and IL-4 expression by CD4^+^ Tconv (YFP^–^) cells (**G** and **H**) and IgG1^+^IgD^–^ B cells (**I** and **J**) from the spleens of mice of the respective treatment group. (**K**) Serum concentrations of Ig IgM, IgA, IgG1, IgG2b, IgG2c, IgG3, and IgE of mice of the respective treatment group. (**L** and **M**) Representative immunofluorescence images of IgG deposit (original magnification, ×200) (**L**) and IgG mean fluorescent intensity (MFI) (**M**) of kidneys of mice of the respective treatment group (*n* = 6 per group). (**N** and **O**) Representative microscopic images of H&E staining (original magnification, ×200) (**N**) and histological scores (**O**) of the skin, lungs, livers, and kidneys of mice of the respective treatment group (*n* = 9). Statistical significance was determined by 1-way ANOVA with Tukey’s multiple comparisons. *P < 0.05, **P < 0.01, ***P < 0.001, ****P < 0.0001.

To assess whether global glutamine utilization or only glutamine-dependent bioenergetic pathways were required to sustain autoimmunity, we initially compared the therapeutic effect of a broad glutamine antagonist 6-diazo-5-oxo-L-norleucine (DON) and of a GLS1-specific inhibitor (CB-839) in the context of Foxp3 deficiency (*Foxp3*^ΔEGFPiCre^*R26*^YFP^ mice). Whereas DON broadly inhibits multiple glutamine-utilizing reactions essential for cellular processes, including synthesis of nucleic acids and proteins and generation of αKG for energy metabolism, CB-839 is a selective inhibitor of the enzyme GLS1, converting glutamine into glutamate. CB-839 treatment results in significant reduction in production of αKG and milder reduction in glutathione and glutamine-fueled nonessential AAs (NEAAs) compared with DON. However, the effect of CB-839 is paradoxical, as it is also reported to induce IFN associated gene expression in addition to its inhibitory effect in context of cancer (36). To evaluate the extent of glutamine-dependent bioenergetics and biosynthetic pathways we treated the Foxp3-deficient mice with DON or CB-839, and we found that DON- but not CB-839–treated mice showed a substantial improvement in overall disease severity, body weight index, and survival, as compared with vehicle-treated (PBS) animals (Figure 1, A and B, and Supplemental Figure 1A; supplemental material available online with this article; https://doi.org/10.1172/jci.insight.200076DS1). Cellular analysis revealed that DON, but not CB-839, reduced the frequency and number of splenic ΔTregs, total CD4^+^ and CD8^+^ T cell number, and activated/memory (CD62L^lo^CD44^hi^) CD4^+^ and CD8^+^ Tconv cell frequency (Figure 1, C–F, and Supplemental Figure 1, B, C, and F). Furthermore, frequency and number of proinflammatory cytokine-producing CD4^+^ Tconv, ΔTreg (IFN-γ and IL-4), and CD8^+^ (IFN-γ) Tconv cells were also reduced upon DON treatment compared with those in vehicle-treated controls (Figure 1, G and H, and Supplemental Figure 1, D–F). Of note, Foxp3-deficient mice exhibit abundant autoantibodies in the serum (13, 37). We observed a significant reduction of splenic IgG1^+^IgD^–^ B cells and serum levels of total IgM, IgA, IgG1, IgG2b, IgG2c, IgG3 Ig and direct IgG deposit within the kidneys of DON-treated mice, as compared with vehicle- or CB-839–treated animals (Figure 1, I–M). Indirect binding assays of serum IgG on ear sections of *Rag1*^KO^ mice revealed that only DON treatment reduced the binding capacities of IgG on ear tissue (Supplemental Figure 1, G and H). To confirm the role of glutamine-dependent bioenergetic and biosynthetic pathways on B cell proliferation and class switching, we stimulated in vitro IgD^+^CD27^–^ naive B cells isolated from WT mice with LPS+IL-4, resiquimod+IL-4, and CD40L+IL-4 in presence or absence of CB-839 and DON. We observed that, while CB-839 mildly reduced proliferation and class switching capacities of B cells in vitro in response to these 3 stimuli, DON treatment resulted in a superior inhibitory effect on B cell proliferation and antibody class switching (Supplemental Figure 1, I–P). Furthermore, DON-treated mice had decreased tissue inflammation (skin, liver, lungs, and kidney) compared with vehicle- and CB-839–treated mice (Figure 1, N and O). Furthermore, the role of glutamine-dependent biosynthetic pathways on inflammatory phenotype was extended in a dextran sodium sulfate–induced (DSS-induced) colitis model in the context of Foxp3-sufficient WT animals. Treatment with DSS in drinking water causes damage to the colonic epithelium, disrupting the mucosal barrier and inducing intestinal inflammation. Interestingly, animals receiving DSS in their drinking water along with i.p. DON treatment showed significantly lower body weight loss and reduced colitis severity (Supplemental Figure 2, A–D). This reduction of the severity of the DSS-induced colitis by DON treatment was associated with significantly lower CD4^+^ and CD8^+^ T cell infiltration and tissue damage in the colons of DON-treated mice, compared with vehicle-treated mice (Supplemental Figure 2, E and F). Additionally, CD4^+^ and CD8^+^ T cells showed lower activation (CD62L^lo^CD44^hi^), higher naive T cell frequency, and reduction in proinflammatory cytokine secretion by CD4^+^ (IFN-γ and IL-4) and CD8^+^ (IFN-γ) Tconv cells (Supplemental Figure 2, G–N). Together, these results show that global blockade of glutamine functions, rather than its bioenergetic roles, support dysregulated autoreactive T and B cells to promote Foxp3 deficiency–mediated disease, suggesting that glutamine-dependent biosynthetic pathways play a predominant role in autoimmunity.

### DON exerts beneficial effects independently of ΔTregs in Foxp3-deficient mice.

**Figure 2:**
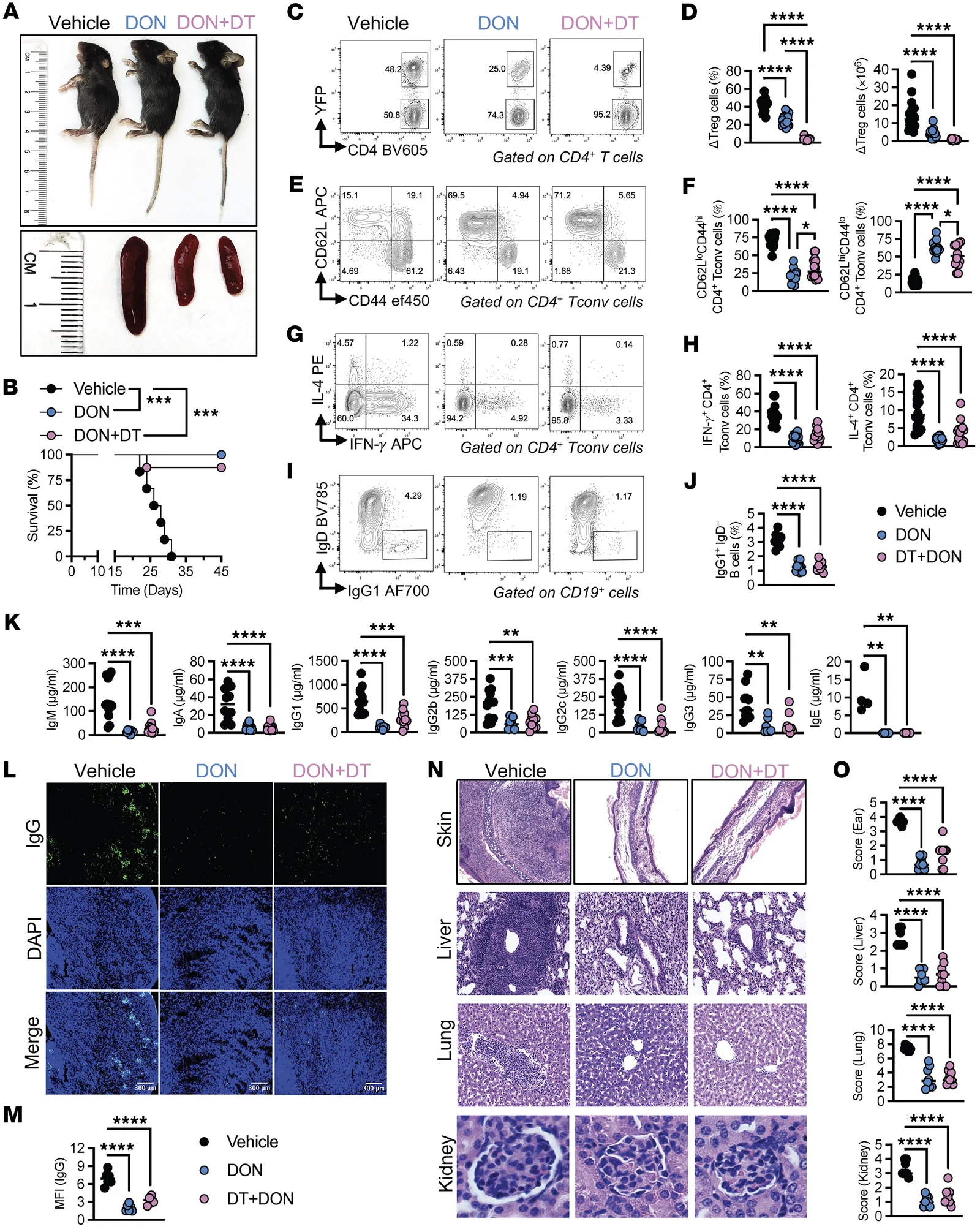
DON exerts beneficial effects independently of ΔTregs in Foxp3-deficient mice. (**A** and **B**) Gross appearance and the respective spleens of 21-day-old *Foxp3*^ΔEGFPiCre^*R26*^YFP/iDTR^ mice treated with vehicle and DON with or without diphtheria toxin (DT) (**A**) and survival over time (**B**). The results represent 1 of the 4 experiments. (**C**–**J**) Representative flow cytometric analysis and frequencies (scatterplots and means) of CD4^+^YFP^+^ ΔTregs (**C** and **D**); CD62L^lo^CD44^hi^, CD62L^hi^CD44^lo^ CD4^+^ Tconv (YFP^–^) cells (**E** and **F**); and IFN-γ and IL-4 expression by CD4^+^ Tconv (YFP^–^) cells (**G** and **H**) and IgG1^+^IgD^–^ B cells (**I** and **J**) from the spleens of mice of the respective treatment group. (**K**) Serum concentrations of Ig IgM, IgA, IgG1, IgG2b, IgG2c, IgG3, and IgE of mice of the respective treatment group. (**L** and **M**) Representative immunofluorescence images of IgG deposit (original magnification, ×200) (**L**) and IgG MFI (**M**) of kidneys of mice of the respective treatment group (*n* = 6 per group). (**N** and **O**) Representative microscopic images of H&E staining (original magnification, ×200) (**N**) and histological scores (**O**) of the skin, lungs, livers, and kidneys of mice of the respective treatment group (*n* = 9 per group). Statistical significance was determined by 1-way ANOVA with Tukey’s multiple comparisons. *P < 0.05, **P < 0.01, ***P < 0.001, ****P < 0.0001.

ΔTregs produced proinflammatory cytokines such as IFN-γ and IL-4 and can potentially behave like pathogenic T cells and contribute to disease due to their potential autoreactivity in periphery of Foxp3-deficient mice. Since in vivo glutamine blockade by DON in Foxp3-deficient mice modulates not only T and B cell responses but also ΔTregs themselves, to evaluate the contribution of ΔTreg reprogramming by DON treatment, we tested the effect of ΔTreg depletion in DON-treated Foxp3-deficient mice (Figure 2). To accomplish ΔTreg depletion, *Foxp3*^ΔEGFPiCre^*R26*^YFP^ and *R26*^iDTR^ mice were crossed, allowing ΔTreg-specific expression of diphtheria toxin receptor (DTR), and upon diphtheria toxin (DT) treatment, ΔTreg depletion occurred. Surprisingly, the effect of DON on the Foxp3 deficiency–mediated disease was preserved even in ΔTreg-depleted mice (Figure 2, A–D, and Supplemental Figure 3A). Furthermore, the effect of DON on CD4^+^ Tconv effector memory frequency and number, Th1/Th2 cytokine (IFN-γ/IL-4) production, CD4^+^ and CD8^+^ naive T cell number, CD8^+^ Tconv effector memory frequency and IFN-γ production were only marginally altered upon ΔTreg depletion (Figure 2, E–H, and Supplemental Figure 3, B–F). Similar we observed a modest effect by DON on IgG1 class switching of B cells, serum Ig levels, autoantibodies against skin antigens and IgG deposit in kidney by DON in presence or absence of ΔTregs was observed (Figure 2, I–M, and Supplemental Figure 3, G and H). Finally, the overall effect of DON on tissue pathology was similar irrespective of the presence of ΔTregs (Figure 2, N and O). These results suggest that the salutary effects of DON on autoreactive and activated T and B cells are independent of ΔTreg metabolic reprogramming in the context of Foxp3 deficiency.

### Glutamine usage blockage alters metabolic landscape.

**Figure 3:**
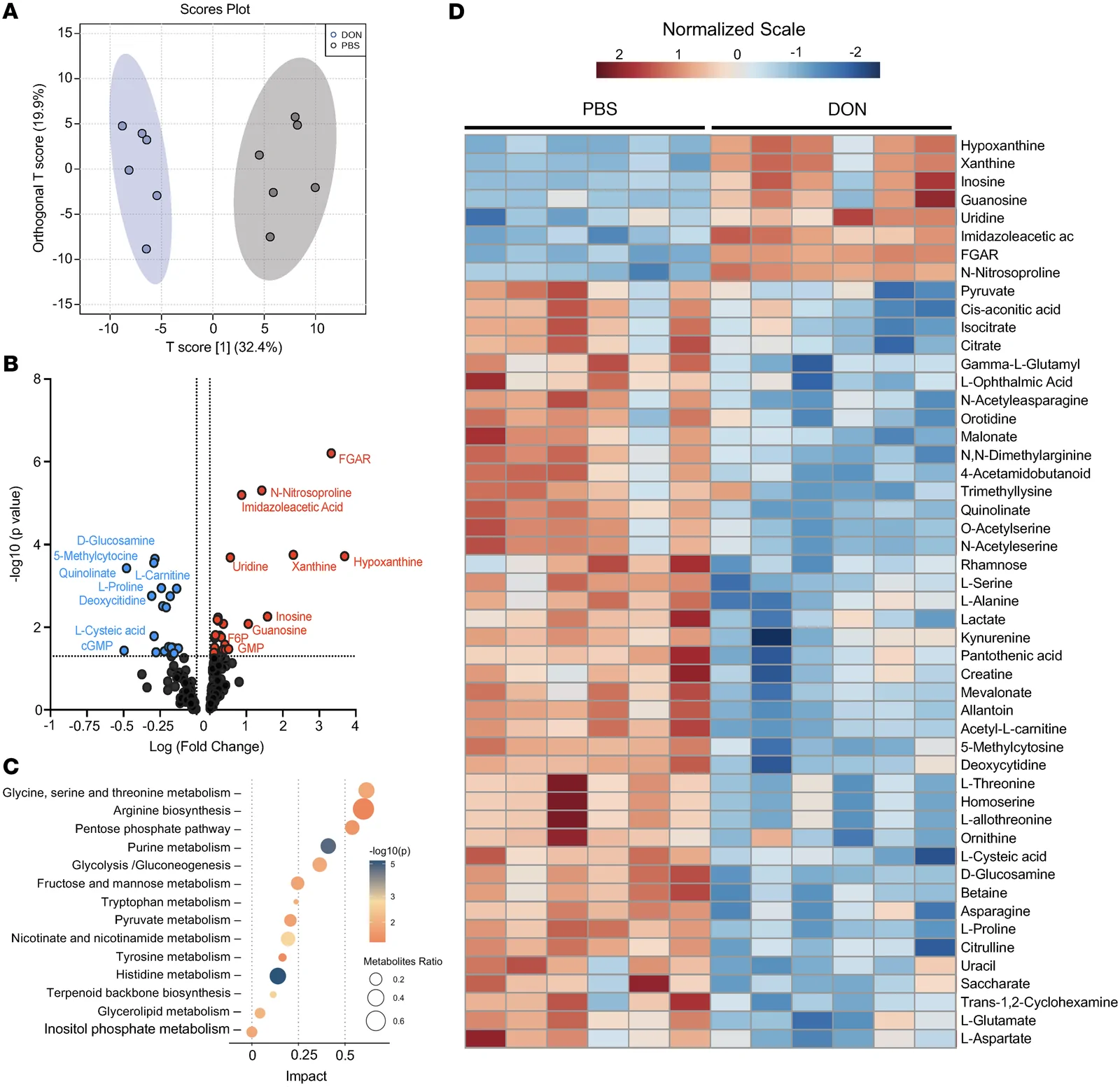
In vivo glutamine blockade alters purine and nonessential amino acid metabolism. (**A**) Principal component analysis (PCA) scores plot showing differentially expressed metabolites in the serum of DON- versus PBS-treated *Foxp3*^ΔEGFPicre^*R26*^YFP^ mice. (**B**) Loading plots showing metabolites upregulated (red) versus downregulated (blue) in the serum of DON-treated *Foxp3*^ΔEGFPicre^*R26*^YFP^ mice. (**C**) Lollipop diagram showing the markedly different KEGG pathways in DON- versus PBS-treated mouse plasma metabolites. (**D**) Heatmap comparing the abundance of plasma metabolites in DON- versus PBS-treated Foxp3-deficient mice. Red and blue indicate increased and decreased metabolite abundance, respectively, in the plasma of DON-treated mice relative to PBS-treated mice.

**Figure 4:**
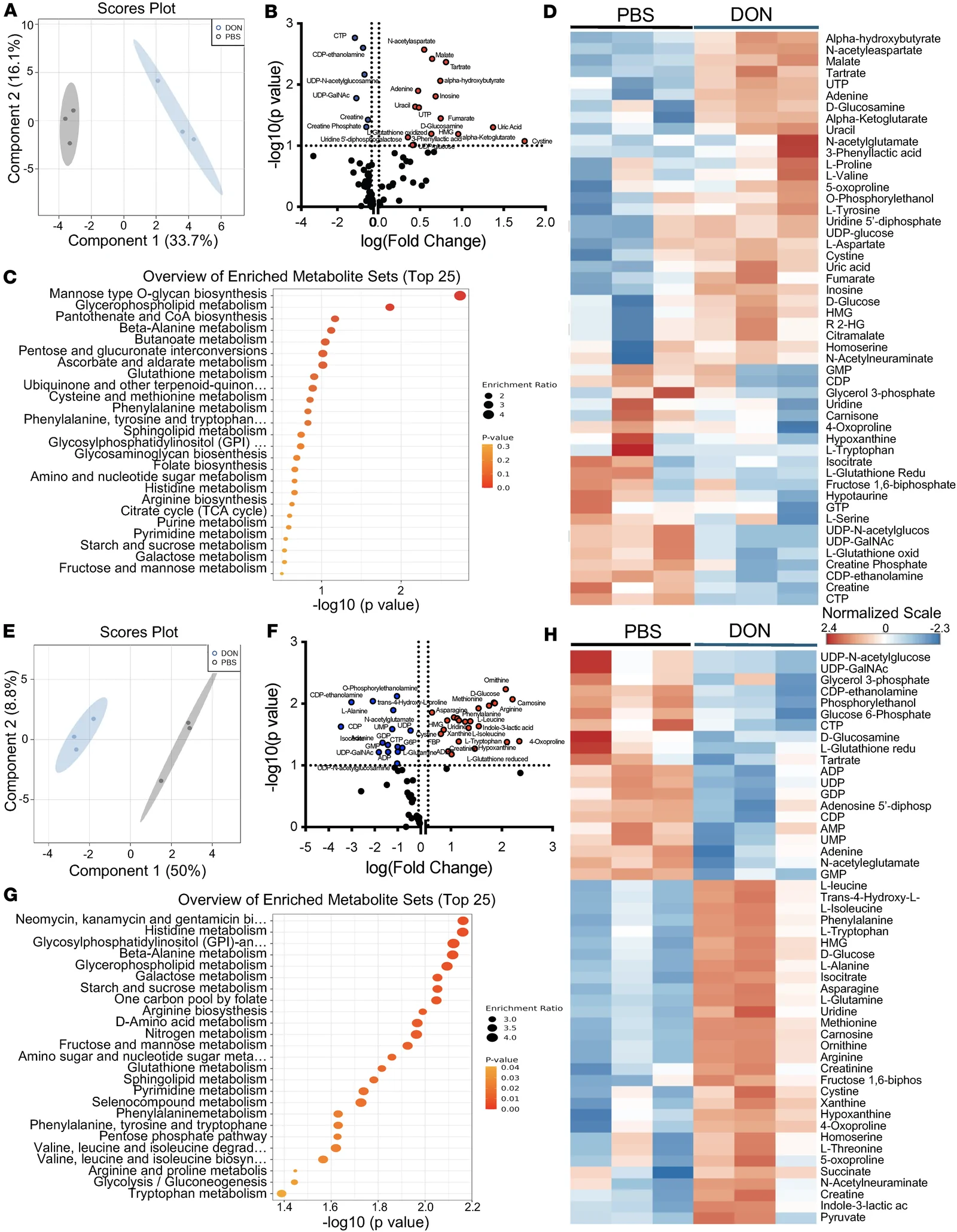
In vivo glutamine blockade in Foxp3-deficient mice alters T and B cell metabolism. (**A**–**D**) PCA plot (**A**), loading plot (**B**), lollipop diagram (**C**), and heatmap (**D**) comparing the metabolites of CD4^+^ T cells isolated from the spleens of DON- versus PBS-treated *Foxp3*^ΔEGFPicre^*R26*^YFP^ mice. (**E**–**H**) PCA plot (**E**), loading plot (**F**), lollipop diagram (**G**), and heatmap (**H**) comparing the metabolites of B cells isolated from the spleen, of DON- versus PBS-treated *Foxp3*^ΔEGFPicre^*R26*^YFP^ mice.

Various intermediate metabolites of glutamine metabolism contribute to different bioenergetic and biosynthetic processes. Glutamine acts as a source of carbon and nitrogen for nucleotide biosynthesis while its metabolite glutamate acts as a substrate for the synthesis of NEAAs and glutathione (38, 39). To further understand how inhibiting glutamine usage improves the Foxp3 deficiency–mediated disease, we analyzed plasma composition of *Foxp3*^ΔEGFPiCre^ mice by liquid chromatography–mass spectrometry (LC-MS) at day 15 after birth following vehicle or DON treatment on day 11 and 14. Using principal component analysis (PCA), we were able to cluster the plasma samples by treatment (Figure 3A). Among 176 metabolites analyzed in vehicle- and DON-treated mouse plasma, 50 showed a marked difference in their relative abundance, represented in volcano plot and heatmap (Figure 3, B–D). A pathway enrichment analysis (using Kyoto Encyclopedia of Genes and Genomes [KEGG]) showed that the most altered metabolites were related to purine and histidine metabolism, suggesting that DON preferentially affects these pathways (Figure 3, C and D). Whereas metabolites related to the purine metabolism (e.g., FGAR, guanosine, inosine, xanthine, hypoxanthine,) were in higher relative abundance, metabolites related to glycolysis (e.g., lactate, pyruvate), TCA cycle (e.g., citrate, isocitrate), and glutamine-dependent AA synthesis (e.g., alanine, aspartate, asparagine, glutamate, proline, ornithine, serine) were in lower relative abundance in DON-treated mice, compared with vehicle-treated controls (Figure 3D). Since DON, but not CB-839, mitigates Foxp3 deficiency–mediated disease, we concluded that the TCA cycle and other glutamine-dependent bioenergetic metabolic pathways do not play a critical role in the in vivo DON effect in Foxp3-deficient mice. Among the glutamine-dependent biosynthetic pathways, inosine and asparagine (ASN) were substantially altered in differential abundance by DON treatment and represent interesting candidates to dampen autoreactive T and B cell responses, respectively (40–43). Analysis of the top 25 compounds corelated with the inosine and asparagine revealed upregulation of inosine metabolites coupled with a decrease in asparagine metabolites in the serum of DON-treated animals (Supplemental Figure 4, A and B). In agreement, metabolomic analysis of CD4^+^ T cells isolated from DON-treated Foxp3-deficient mice showed a differential metabolomic profile compared with that of CD4^+^ T cells isolated from PBS-treated Foxp3-deficient mice, including higher inosine level and enriched purine metabolic pathway activity in CD4^+^ T cells from DON-treated mice (Figure 4, A–D). These results confirm the direct role of inosine on DON-mediated T cell suppression. Additionally, metabolomics analysis of B cell isolated from DON-treated mice presented altered metabolomic profile, with enrichment of metabolites related to AA metabolism (Figure 4, E–H). Interestingly, there was a trend of higher asparagine level within B cell as opposed to serum asparagine level (Figure 4H). This observation reflects reduced utilization of asparagine by B cells upon DON treatment. These results support the direct role of glutamine on T and B cells mediated by inosine and asparagine, respectively.

### Inhibition of glutamine usage controls activation of autoreactive T and B cells in inosine- and asparagine-dependent manners, respectively.

**Figure 5:**
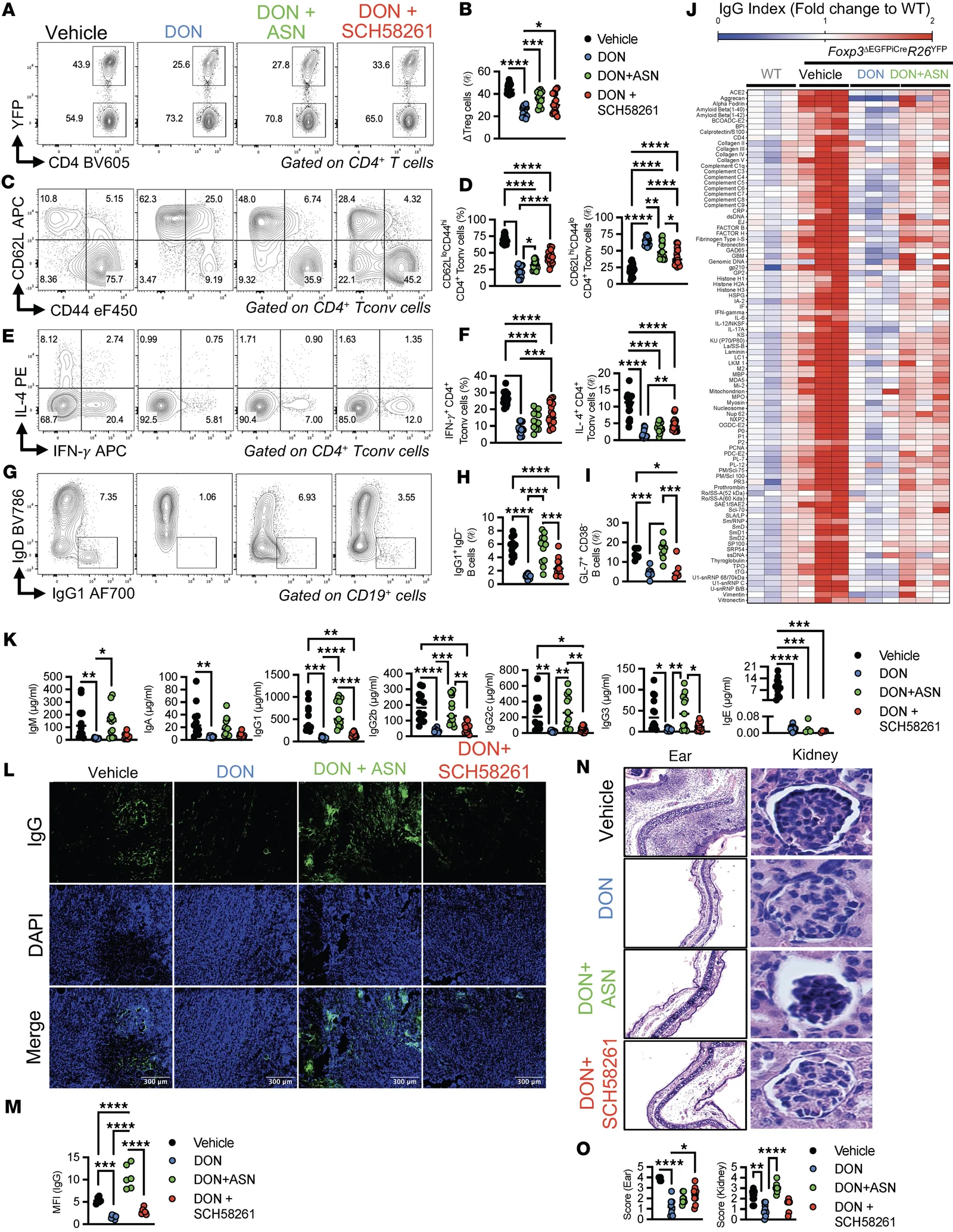
Inhibition of glutamine usage controls activation of autoreactive T and B cells in inosine- and asparagine-dependent manners, respectively. (**A**–**I**) Representative flow cytometric analysis and frequencies (scatterplots and means) of CD4^+^YFP^+^ ΔTregs (**A** and **B**); CD62L^lo^CD44^hi^, CD62L^hi^CD44^lo^ CD4^+^ Tconv (YFP^–^) cells (**C** and **D**); IFN-γ and IL-4 expression by CD4^+^ Tconv (YFP^–^) cells (**E** and **F**), IgG1^+^IgD^–^ B cells (**G** and **H**), and GL-7^+^CD38^–^ germinal center B cells (**I**) from the spleens of mice treated with vehicle, DON, DON+ASN, or DON+SCH58261. (**J**) Heatmap of IgG autoantigen array analysis in the serum of untreated age-matched WT and *Foxp3*^ΔEGFPicre^*R26*^YFP^ mice treated with vehicle, DON, or DON+ASN. Fold change values for each group were calculated by normalizing with the values of WT mice. The observed fold change values are color-coded per the legend at the top of the heatmap. (**K**) Serum concentrations of Ig IgM, IgA, IgG1, IgG2b, IgG2c, IgG3, and IgE of mice of the respective treatment group. (**L** and **M**) Representative immunofluorescence images of IgG deposit (original magnification, ×200) (**L**) and mean fluorescence intensity (**M**) of kidneys of mice of the respective treatment group (*n* = 6). (**N** and **O**) Representative microscopic images of H&E staining (original magnification, ×200) (**N**) and histological scores (**O**) of the skin and kidneys of mice of the respective treatment group (*n* = 9). Statistical significance was determined by 1-way ANOVA with Tukey’s multiple comparisons. *P < 0.05, **P < 0.01, ***P < 0.001, ****P < 0.0001.

Inosine binds the adenosine (A2A) receptor expressed on effector T cells to prevent Th1/Th2 skewing of T cells, while asparagine can be taken up directly by immune cells, particularly germinal center B cells (43–45). To confirm the role of altered inosine and asparagine levels on T and B cell activity following DON treatment, we treated *Foxp3*^ΔEGFPiCre^*R26*^YFP^ mice with DON in combination with an A2A receptor antagonist (SCH58261) or with asparagine supplementation. Cotreatment of DON with SCH58261 but not asparagine partially reversed the effect of DON on CD4^+^ Tconv effector memory frequency and number, Th1/Th2 cytokine (IFN-γ/IL-4) production by Tconv cells and ΔTregs, CD4^+^/CD8^+^/CD4^+^ effector T cell memory, and naive T cell number, CD8^+^ T cell number and IFN-γ production (Figure 5, A–F, and Supplemental Figure 5, A–H). In contrast, while concurrent asparagine supplementation with DON marginally affected T cell responses, it reversed the effect of DON on B cell activation and autoreactivity, including restoration of IgG1 class switching of B cells, germinal center B cell formation, IgG/IgM autoreactivity against self-antigens, high serum Ig levels, and IgG antibody deposit in kidney (Figure 5, G–M, and Supplemental Figure 5I). As a result, concurrent A2A receptor blockade reversed the beneficial effect of DON on skin inflammation in the context of asparagine supplementation restoration of IgG deposit in kidney resulted in increased severity of kidney pathology (Figure 5, N and O). By comparing the in vitro capacities of naive B cells isolated from WT mice to proliferate and secrete IgG1 upon LPS+IL-4 stimulation in presence or absence of DON and asparagine in asparagine low (DMEM) and rich (RPMI) medium, we confirmed that asparagine is a critical NEAA for B cell proliferation and IgG1 production that can partially reverse the effect of DON (Supplemental Figure 5, J–L). Overall, the effects of A2A receptor blockade and asparagine supplementation in the presence of DON on T and B cells suggest that DON alters glutamine dependent biosynthetic pathways to inhibit T and B cell functions, respectively, via inosine and asparagine to mitigate the Foxp3 deficiency–mediated disease.

### Asparagine exclusively controls activation of B cell in Foxp3 deficiency–mediated disease.

**Figure 6:**
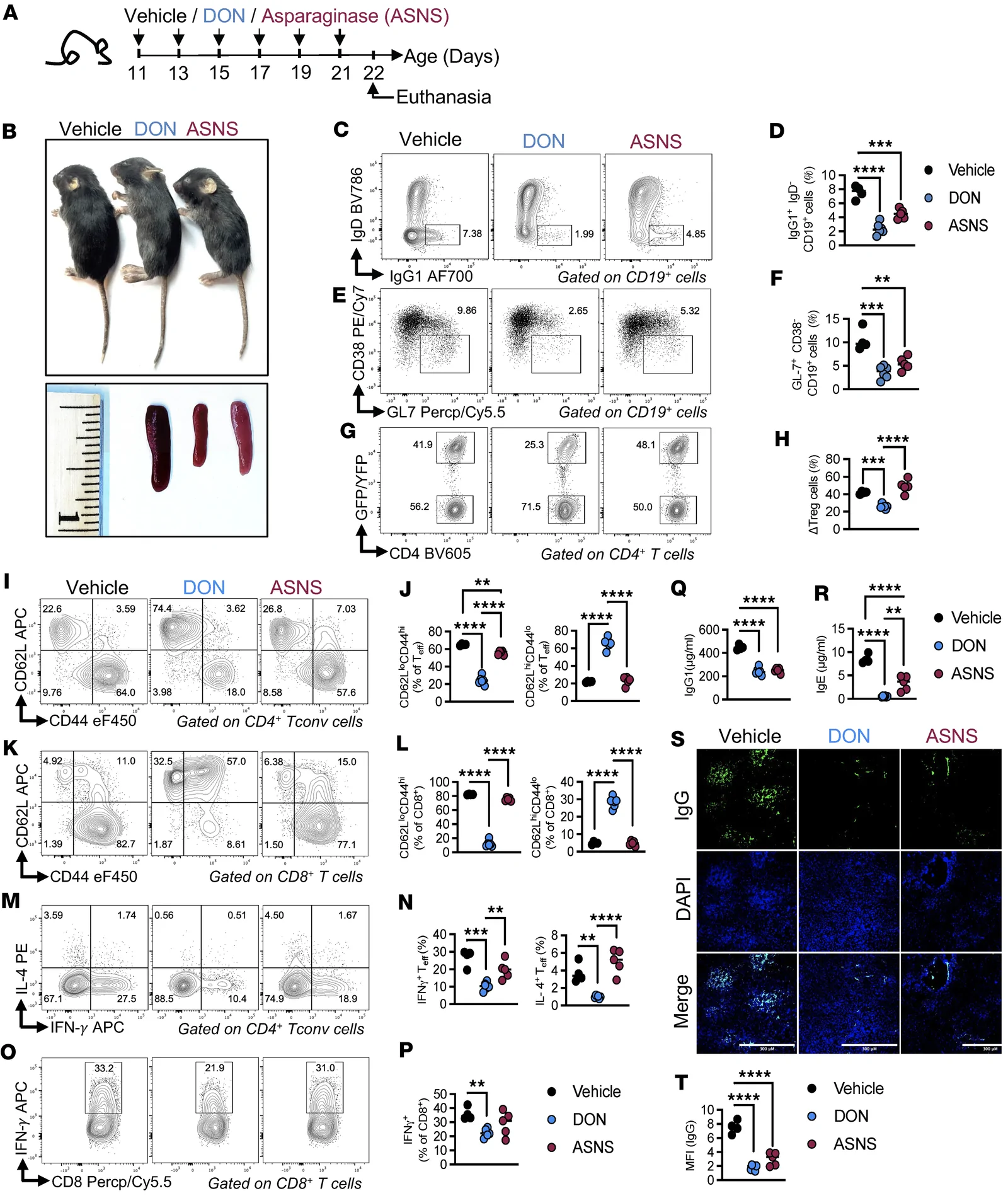
Asparagine exclusively controls B cell dysregulation in Foxp3 deficiency–mediated disease. (**A** and **B**) Treatment strategy (**A**) and gross appearance and the respective spleens of 21-day-old *Foxp3*^ΔEGFPiCre^*R26*^YFP^ mice treated with vehicle, DON, or asparaginase (ASNS) (**B**). The results represent 1 of the 3 experiments. (**C**–**P**) Representative flow cytometric analysis and frequencies (scatterplots and means) of IgG1^+^IgD^–^ B cells (**C** and **D**), GL-7^+^CD38^–^ B cells (**E** and **F**), CD4^+^YFP^+^ ΔTregs (**G** and **H**), CD62L^lo^CD44^hi^, CD62L^hi^CD44^lo^ CD4^+^ Tconv (YFP^–^) cells (**I** and **J**), CD62L^lo^CD44^hi^, CD62L^hi^CD44^lo^ CD8^+^ Tconv (YFP^–^) (**K** and **L**), IFN-γ and IL-4 expression by CD4^+^ Tconv (YFP^–^) cells (**M** and **N**), and IFN-γ expression by CD8^+^ Tconv (YFP^–^) cells (**O** and **P**) from the spleens of mice of the respective treatment group. (**Q** and **R**) Serum concentrations of Ig IgG1 (**Q**) and IgE (**R**) of mice of the respective treatment group. (**S** and **T**) Representative immunofluorescence images of IgG deposit (original magnification, ×200) (**S**) and IgG mean fluorescent intensity (MFI) (**T**) of kidneys of mice of the respective treatment group (*n* = 6) per group. Statistical significance was determined by 1-way ANOVA with Tukey’s multiple comparisons. *P < 0.05, **P < 0.01, ***P < 0.001, ****P < 0.0001.

Next, we wanted to assess the selective dependence of autoreactive B cell activation on asparagine in the context of Foxp3 deficiency using the asparagine-depleting enzyme asparaginase (ASNS). We observed that treatment of *Foxp3*^ΔEGFPiCre^*R26*^YFP^ mice with ASNS has mild effect on gross representation and splenomegaly compared with treatment with DON (Figure 6, A and B). Consistent with asparagine supplementation with DON (Figure 5), depletion of asparagine with ASNS significantly reduced the IgG1 class switching of B cells and germinal center (GC) formation similar to DON (Figure 6, C–F). Notably, in contrast to DON treatment, ASNS treatment did not reduce the frequency of ΔTregs (Figure 6, G and H). Furthermore, unlike DON treatment, ASNS treatment also failed to control CD4^+^/CD8^+^ Tconv effector memory cell frequency, Th1/Th2 cytokine (IFN-γ/IL-4) production by CD4^+^ Tconv cells, and IFN-γ production by CD8^+^ Tconv cells (Figure 6, I–P). Furthermore, like its effect on B cell class switching and GC formation ASNS treatment resulted in significantly reduced serum IgG1 and IgE levels and IgG deposit in kidney, comparable to DON treatment (Figure 6, Q–T). These results suggest that alteration in asparagine bioavailability upon DON treatment results specifically in inhibition of B cell activation in the context of Foxp3 deficiency.

## Discussion

Upon tolerance breakdown, immune cells continuously proliferate and produce inflammatory mediators (cytokines, chemokines, antibodies, etc.). To exert these functions, lymphocytes undergo extensive metabolic reprogramming. Recent understanding of metabolic reprogramming of immune cells in inflammatory conditions has paved the way for the development of novel therapeutics against inflammatory/autoimmune diseases. Many antiinflammatory drugs, such as metformin, rapamycin, dimethyl fumarate, and methotrexate, can also directly effect immune cell metabolism by acting on different metabolic pathways, including glucose metabolism, mTORC1, pentose phosphate pathway, fatty acid metabolism, and nucleotide biosynthesis (46). Interestingly, among different metabolic pathways, glutamine metabolism is still an underappreciated target for therapeutic control of immune cell activation. Breakdown of glutamine in proliferating cells serves as a carbon and nitrogen source to fuel bioenergetic and biosynthetic pathways. Deamination of glutamine to glutamate by GLS1/2 replenishes metabolic intermediate via TCA cycle for energy metabolism (47). Additionally, glutamine is involved in de novo synthesis of various metabolites such as nucleotides, NEAAs, and glutathione (48). These molecules serve as a substrate for synthesis of effector molecules (cytokines, chemokines, antibodies etc.) required for T and B cell proliferation, survival, and effector function. Given the reliability of autoreactive T and B cells on glutamine-dependent biosynthetic pathways, these pathways can be targeted for treatment of inflammatory disorders.

In this study we evaluated the contribution of dysregulated glutamine-dependent metabolic pathways used by autoreactive T and B cells to promote autoimmunity. Our results demonstrate that glutamine dependent biosynthetic but not bioenergetic pathways are essential to T and B cell activation/proliferation, inflammatory cytokines, and autoantibody production in the context of Foxp3 deficiency and a DSS-induced colitis model. Inhibition of GLS1 by using CB-839 to target bioenergetic pathways had little to no effect on the severity of autoimmune disease mediated by Foxp3 deficiency, while targeting both glutamine-dependent bioenergetic and biosynthetic pathways using a broad inhibitor of glutamine utilizing enzyme DON resulted in overall improvement in the disease.

Plasma metabolite analysis revealed significant alteration in key metabolites (purine, pyrimidine, asparagine, etc.) related to glutamine metabolism in DON-treated mice, suggesting the involvement of glutamine dependent biosynthetic pathways. Inhibition of glutamine-to-glutamate conversion resulted in higher purine and pyrimidine nucleotides, particularly inosine. Previous studies have demonstrated the antiinflammatory effect of inosine, as inosine treatment reduced the production of proinflammatory cytokines and chemokines and attenuated the course of chronic autoimmune inflammatory diseases, including murine type 1 diabetes and experimental colitis (49, 50). Mechanistically, inosine binds the A2A receptor expressed on effector T cells to inhibit the Th1/Th2 polarization (41, 50, 51). Furthermore, one study identified reduced levels of inosine in Foxp3-deficient mice and found that replenishment of inosine resulted in improvement of the scurfy phenotype (41). We confirmed the role of elevated inosine levels on DON-mediated amelioration of disease by utilizing A2A receptor antagonist SCH58261. Coadministration of DON along with SCH58261 resulted in partial reversal of DON-mediated effects on T cells.

Glutamate also acts as a nitrogen donor for the synthesis of NEAA. Inhibition of glutamine metabolism by DON resulted in reduced NEAA abundance. Among various NEAAs that were substantially reduced in the plasma of DON-treated animals, asparagine was the most promising candidate, given its role in the B cell activation and germinal center formation (43). Our results indicated asparagine as a key regulator of B cell homeostasis specially in autoantibody production. In this study, we showed that glutamine-dependent asparagine availability in Foxp3-deficient mice is critical for B cell autoreactivity and autoantibody production, and by targeting glutamine-dependent asparagine synthesis, we reduced the B cell autoreactivity in the context of Foxp3 deficiency, suggesting a critical role of asparagine in B cell dysregulated responses in autoimmune settings.

DON has been studied for decades as a potential anticancer therapeutic. Our result showed that it can be useful for the treatment of inflammatory disorders and autoimmunity largely owing to its effect on glutamine dependent biosynthetic pathways responsible for generation of metabolic intermediates required for activation of T and B cells. More broadly, our studies uncover the possibility of combinatorial interventions that target distinct metabolic pathways to restore immune tolerance in a variety of autoimmune and immune dysregulatory diseases.

## Methods

### Sex as a biological variable.

Both male and female mice were included in all experiments. Animals were age matched and distributed across experimental groups without sex-based preselection.

### Animals and treatment.

C57BL/6 WT and *Rag1*^KO^ mice were purchased from The Jackson Laboratory. The *Foxp3*^ΔEGFPiCre^*R26*^YFP^ strain was generated as described previously (9)*, and Foxp3*^ΔEGFPiCre^*R26*^YFP/iDTR^ mice were generated by crossing *Foxp3*^ΔEGFPiCre^*R26*^YFP^ mice with *R26*^iDTR^ mice (The Jackson Laboratory). Mice were treated with DON, CB-839, SCH58261, asparagine, and asparaginase (3 μg/g, 12.5 μg/g, 6 μg/g, 24 μg/g, and 5U/g of bodyweight, respectively), i.p. for every alternate day from day 11 to 21 for analysis and continued with the same treatment strategy up to day 45 for survival analysis. *Foxp3*^ΔEGFPiCre^*R26*^YFP/iDTR^ mice were treated with DT (0.5 μg/mouse on day 11 and 0.25 μg/mouse from day 12 onward) i.p. for every alternate day up to day 21. Animals were sacrificed at day 22 for analysis, and serum, spleens, ears, lungs, livers, and kidneys were collected for autoantibody array, antibody ELISA, flow cytometry, histopathology, and immunohistochemistry. Animals were kept under specific pathogen–free conditions for the entire duration of experiment and used in accordance with institutional animal research committees at the Boston Children’s Hospital.

### Reagents.

DON (catalog D2141), DT (catalog D0564), and L-asparagine (catalog A0884) were acquired from Sigma-Aldrich. SCH58261 was purchased from Tocris (catalog 2270). CB-839 was purchased from Cayman Chemical Company (catalog 22038), and L-asparaginase was purchased from Medchemexpress (catalog HY-P1923). All the reagents were diluted and stored according to manufacturer’s instruction.

### B cell class switching and proliferation assay.

IgD^+^CD27^–^ naive B cells were purified from WT mice by using the Dynabeads Mouse CD43 kit (Invitrogen) according to the manufacturer’s instructions. Cell Trace Violet–labeled 5 × 10^4^ naive B cells were cultured in RPMI medium supplemented with 10% FBS, 1 mM sodium pyruvate,10 mM HEPES, 100 IU/mL penicillin/streptomycin, and 50 μM 2-mercaptoethanol, aspartate (150 μM), glutamate (140 μM), and GlutaMAX (2mM) for 48 hours in presence or absence of LPS (10 μg/mL)/resiquimod (5 μg/mL)/CD40L (5 μg/mL) with IL-4 (50 ng/mL) in a 96-well plate. DMEM without NEAA was used as ASN-free medium wherever indicated. Cells were treated with DON or ASN or a combination, as indicated in the figures. For proliferation assay, after Fc block, cells were stained with viability dye and CD19. For class switching assay, splenocytes or cultured cells were incubated with monoclonal anti-IgE antibody along with Fc block followed by staining with viability dye and surface markers against CD19 and IgD and later stained intracellularly with anti-IgG1, IgM, and IgE antibodies.

### Flow cytometry.

Cells were stained as previously described (9). Viability dye and various anti-mouse antibodies against CD44 (eBioscience), CD90.1, CD4, CD8a, CD62L, IFN-γ, IL-4, CD19, IgD, IgG1, and IgM, along with Fc block (CD16/32) (Biolegend), were used. Briefly, cells were incubated for 10 minutes with Fc block followed by viability dye and surface markers for 20 minutes. For intracellular markers, after surface staining, cells were fixed and permeabilized with BD Cytofix/Cytoperm buffer for 30 minutes and incubated overnight with antibodies against intracellular markers. For cytokine detection cells were prestimulated with 50 ng/mL phorbol myristate acetate, 500 ng/mL ionomycin, and 10 μg/mL brefeldin A for 4 hours at 37°C in RPMI medium with 10% FBS. After staining cells were acquired using BD Fortessa with DIVA software (BD Biosciences) and analyzed using FlowJo software. All the antibodies used for flow cytometry are listed in the Supplemental Table 1.

### Histopathology.

Ear, kidney, lung and liver sections were stained with hematoxylin and eosin. Bright-field images at ×200 magnification were taken on an EVOS M700 (Invitrogen) microscope. Histological scores were made by a blinded observer at an average of 3 different fields per each section. Ear inflammation was scored as follows: 0, no inflammation; 1, mild inflammation associated with infiltration of a few cells; 2, moderate inflammation associated with mild infiltration; 3, severe inflammation associated with large infiltration of cells and mild skin dryness; and 4, very severe inflammation associated with skin dryness and cartilage erosion. The glomerular lesions were graded on a scale of 0–3 as previously described (52). Histopathologic findings in renal vascular lesions were graded on a scale of 0–3: 0, normal; 1, mild (perivascular cell infiltration); 2, moderate (destruction of arterial wall); and 3, severe (myointimal thickening). Lung inflammation was scored separately for cellular infiltration around blood vessels and airways, as follows: 0, no infiltrates; 1, few inflammatory cells; 2, a ring of inflammatory cells 1-cell layer deep; 3, a ring of inflammatory cells 2 to 4 cells deep; and 4, a ring of inflammatory cells more than 4 cells deep. A composite score was determined by adding the inflammatory scores for both vessels and airways. Liver inflammation was scored at portal areas as follows: 0, no inflammatory cells; 1, mild, scattered infiltrates; 2, moderate infiltrates occupying <50% of the portal areas; 3, extensive infiltrates in the portal areas; and 4, severe, with infiltrates completely packing the portal area and spilling over into the parenchyma.

### Immunohistochemistry.

Kidney cryosections were fixed with acetone followed by blocking with 2% BSA and stained with fluorochrome-conjugated anti-mouse IgG antibody for evaluating direct antibody deposit in kidney. Ear cryosections from *Rag1*^KO^ mice were used to detect autoreactive antibodies against skin antigens. After fixation and blocking, ear samples were incubated with serum (1:30) from 22-day-old mice of the respective genotypes and treatments for 1 hour followed by incubation with anti-mouse IgG antibody for 30 minutes. Finally, sections were mounted using VECTASHIELD PLUS antifade mounting medium with DAPI (Vector Laboratories), and images were collected using a fluorescence microscope and processed using ImageJ software (NIH). Final scores reflected averages of scores from 3 different ×200 fields per tissue per mouse.

### Antibody ELISA.

ELISA was performed to detect total IgE/IgG1 antibodies in the culture supernatant as previously described (53). ELISA plates coated overnight with capture antibodies were blocked with 2% BSA solution. Supernatant samples and standards were incubated at room temperature for 2 hours. Next, biotin-conjugated secondary antibodies were added for 1 hour followed by incubation with avidin HRP solution (Biolegend) for 45 minutes. Finally, wells were developed by using TMB substrate (BD Biosciences), and the reaction was stopped using 1 M HCl and read at 450 nm using a spectrophotometer.

### Mouse Ig isotyping panel detection.

Concentration of IgG1, IgG2b, IgG2c, IgG3, IgA, IgM, and IgE Ig isotypes in the serum of different groups was measured using a bead-based LEGENDplex Mouse Immunoglobulin Isotyping Panel (Biolegend) according to the manufacturer’s instruction.

### Metabolomics analysis by LC-MS.

*Foxp3*^ΔEGFPiCre^*R26*^YFP^ mice received i.p. injections of DON (0.3 mg/kg) or PBS on days 11 and 14. On day 15, whole blood was collected in K2-EDTA tubes and centrifuged at 1,500*g* for 15 minutes at 4°C. Metabolite extraction was then performed on the plasma fraction by adding 1 mL of –20°C methanol and chloroform solvent (60:40), vortexing for 15 minutes at 4°C, and centrifuging at 21,000*g* for 15 minutes at 4°C. The supernatant containing polar metabolites was dried using a 4°C CentriVap SpeedVac (Labconco). Dried metabolite samples were resuspended in 50:50 acetonitrile/water for LC-MS analysis. Metabolites were resolved on a Vanquish U-HPLC system coupled to a Q Exactive HF-X hybrid quadrupole-orbitrap mass spectrometer (Thermo Fisher) with a HESI source operating in negative ion mode. The analytes were separated by using an iHILIC column (5 mm, 150 × 2.1 mm I.D., HILICON) coupled to a Thermo Scientific SII UPLC system. The iHILIC column was used with the following buffers: A, water with 20 mM ammonium carbonate with 0.1% ammonium hydroxide, and B, acetonitrile. The Vanquish U-HPLC was run at a flow rate of 0.150 mL/min: 0–23 minutes linear gradient from 95% B to 5% B; 23–25 minutes hold at 5% B, to waste from 25–25.5 minutes gradient to 95% B at 0.20 mL/min, 25.5–32.5 minutes hold at 95% B, and finally 32.5–33 minutes 95% B at 0.15 mL/min. For cellular metabolomics FACS-sorted CD4^+^ Tconv cells or B cells from the spleens of *Foxp3*^ΔEGFPiCre^*R26*^YFP^ mice treated with DON or PBS were used. MS data targeted feature extraction and quantification were performed on TraceFinder v4.1 (Thermo Fisher). Peak area integration and metabolite identification were performed using accurate mass and retention time curated with in-house standard library compounds. Data were normalized by cell number, and internal standard D8-phenylalanine was spiked during metabolite extraction to account for variations introduced during sample handling, preparation, and injection. MetaboAnalyst 5.0 platform was used for metabolomics data analysis.

### DSS-induced colitis model.

Colitis was induced by oral administration of DSS MW ca 40,000, Fisher Scientific). After acclimatization, 8-week-old C57BL/6 mice were randomly assigned to 2 groups: 3.5% (w/v) DSS-treated group and 3.5% DSS + DON–treated group. For the DON-treated group, mice were treated i.p. with DON (30 μg/mice) from day 0 to day 6 every alternate day. Body weight, stool softness, and blood in the rectum or stool were recorded daily from day 0 to day 6. Clinical scoring of classical IBD was performed as follows: score 0, healthy; 1, weight lose; 2, weight lose and loose stools (pasty and semiformed stools that did not adhere to the anus); 3, weight lose, loose stools, and positive occult blood in stools; 4, weight lose, diarrhea (liquid stools that adhere to the anus), and gross bleeding in stools. Mice were sacrificed at day 6 and tissue samples were harvested.

### Isolation of lamina propria cells from colon.

Colons were isolated from each animal. Each colon was placed on moistened paper towel, and solid stool was extracted by applying mild pressure to the bowel wall with the blunt end of scissors or forceps. Colon were opened longitudinally, and residual fecal waste was removed by vortexing in cold PBS. Cleaned colon was placed in prewarmed cRPMI with EDTA (5 mM) for 15–20 minutes at 37°C in a shaking incubator to remove intraepithelial lymphocytes. Next, colon was washed with PBS and chopped into small pieces and transferred to prewarmed cRPMI with collagenase from *Clostridium histolyticum* (0.25 mg/mL) and kept at 37°C for 45 minutes. Digested tissues were passed through 70 μM cell strainers to get single-cell suspension. Mononuclear cells were isolated by resuspending the cell pellet into 40% Percoll v/v (Sigma-Aldrich) in PBS and centrifuged in a swing bucket centrifuge at 590*g* for 20 minutes without brake. Finally, the cell pellet was washed with PBS and used for flow cytometric analysis.

### Autoantibody array.

Autoantibody autoantigen (anti-mouse IgG, and anti-mouse IgM) screening against 90 autoantigens in the serum samples was performed by Core Facility of the University of Texas Southwestern Medical Center as previously described (54).

### Statistics.

Statistical analysis were performed using GraphPad Prism (11.0.0). Data are presented as mean ± SEM unless otherwise stated. Comparisons between 2 groups were performed using an unpaired, 2-tailed Student’s *t* test. For comparisons among multiple groups, 1-way ANOVA followed by Tukey’s multiple-comparisons test was used. For experiments involving 2 independent variables, 2-way ANOVA with Geisser-Greenhouse correction was applied. A *P* value of less than 0.05 was considered statistically significant.

### Study approval.

All animal experiments were performed in accordance with protocols approved by the Institutional Animal Care and Use Committee of Boston Children’s Hospital.

### Data availability.

Data and supporting data values can be made available from the corresponding author upon request.

## Author contributions

MAZ, MH, and LMC designed experiments. MAZ, CNHM, JB, YZ, XL, SJ, YEF, VC, PG, KK, and LMC performed experiments and analyzed data. PG and KK analyzed metabolomic results. MAZ and LMC made the figures and wrote the manuscript.

## Conflict of interest

The authors have declared that no conflict of interest exists.

## Funding support

This work is the result of NIH funding, in whole or in part, and is subject to the NIH Public Access Policy. Through acceptance of this federal funding, the NIH has been given a right to make the work publicly available in PubMed Central.

- This work was supported by NIH National Institute of Allergy and Infectious Diseases grant R01AI153174 (to LMC).

## Supplementary Material

Supplemental data

Supporting data values

## Version 1 09/08/2026

Electronic publication
