# Supplementary material for: Glutamine-dependent biosynthetic pathways fuel autoreactive T and B cells in Foxp3 deficiency–mediated disease
Source: JCI Insight. 2026 Sep 8;11(17):e200076. doi: 10.1172/jci.insight.200076 (PMC13564079; doi:10.1172/jci.insight.200076)
Supplement: Supplementary file 1 [file jciinsight-11-200076-s105.pdf]

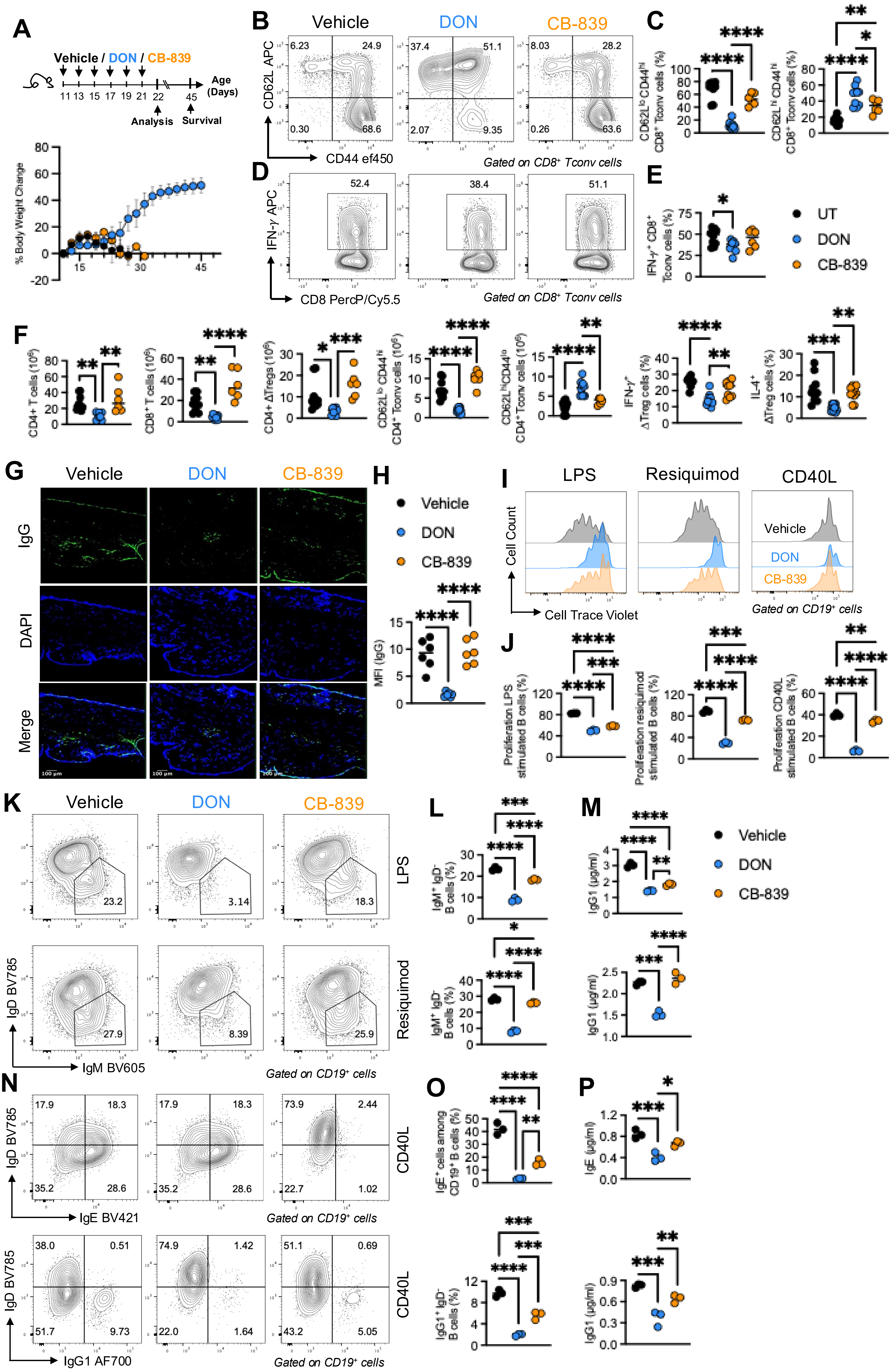

A
Vehicle / DON / CB-839
11 13 15 17 19 21 22 45
Age (Days)
Analysis
Survival
% Body Weight Change
B
Vehicle
DON
CB-839
CD62L APC
CD44 ef450
Gated on CD8⁺ Tconv cells
C
D
IFN-γ APC
CD8 PercP/Cy5.5
E
UT
DON
CB-839
F
G
Vehicle
DON
CB-839
IgG
DAPI
Merge
H
Vehicle
DON
CB-839
MFI (IgG)
I
LPS
Resiquimod
CD40L
Cell Count
Cell Trace Violet
Gated on CD19⁺ cells
J
K
L
M
IgD BV785
IgM BV605
N
IgE BV421
IgG1 AF700
O
P

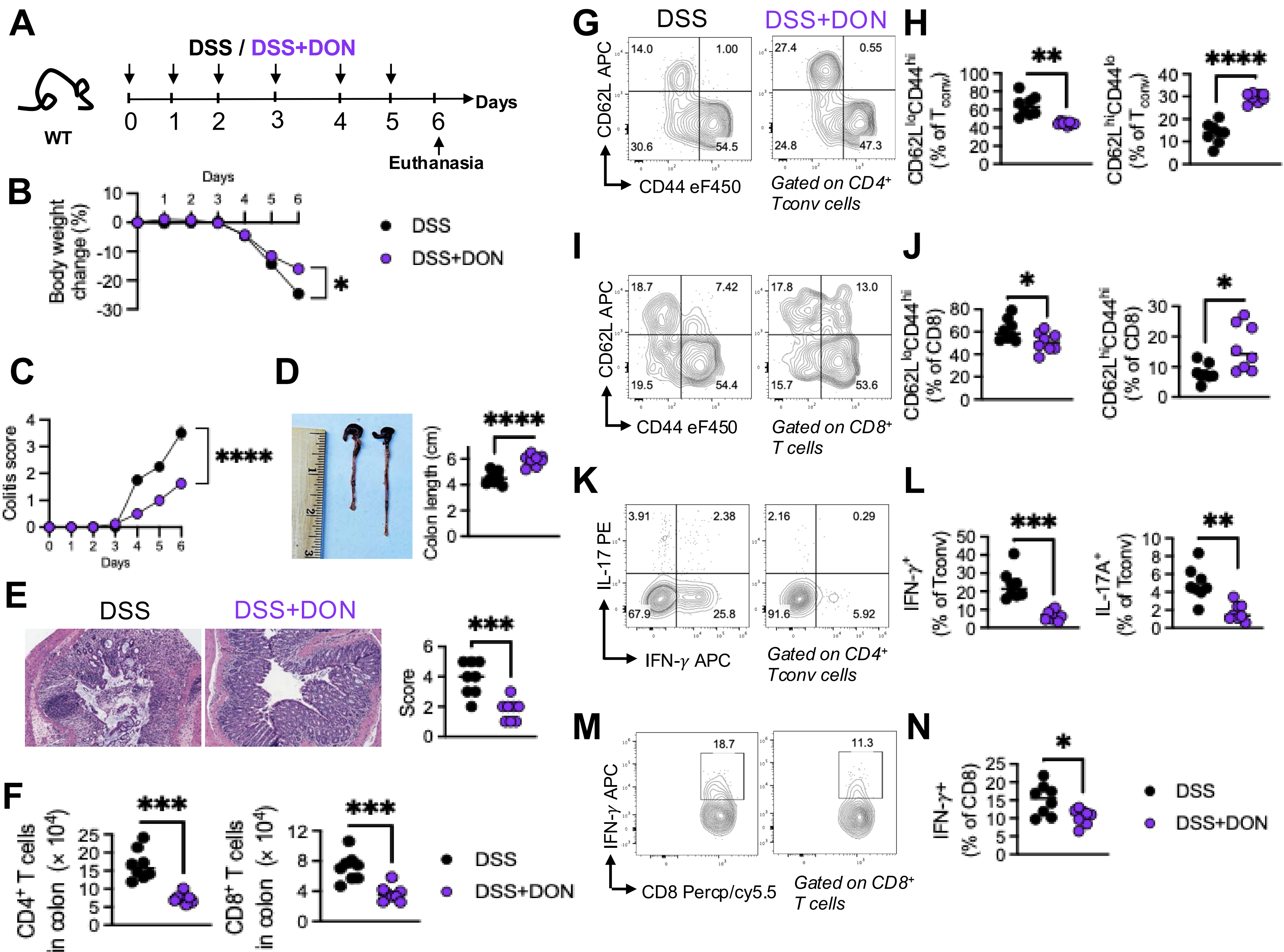

A
DSS / DSS+DON
WT
Days
Euthanasia
B
Body weight change (%)
Days
DSS
DSS+DON
C
Colitis score
Days
D
Colon length (cm)
E
DSS
DSS+DON
Score
F
CD4+ T cells in colon (x 10^4)
CD8+ T cells in colon (x 10^4)
G
DSS
DSS+DON
CD62L APC
CD44 eF450
Gated on CD4+ Tconv cells
H
CD62L^lo CD44^hi (% of Tconv)
CD62L^hi CD44^lo (% of Tconv)
I
CD62L APC
CD44 eF450
Gated on CD8+ T cells
J
CD62L^lo CD44^hi (% of CD8)
CD62L^hi CD44^hi (% of CD8)
K
IL-17 PE
IFN-γ APC
Gated on CD4+ Tconv cells
L
IFN-γ+ (% of Tconv)
IL-17A+ (% of Tconv)
M
IFN-γ APC
CD8 Percp/cy5.5
Gated on CD8+ T cells
N
IFN-γ+ (% of CD8)
DSS
DSS+DON

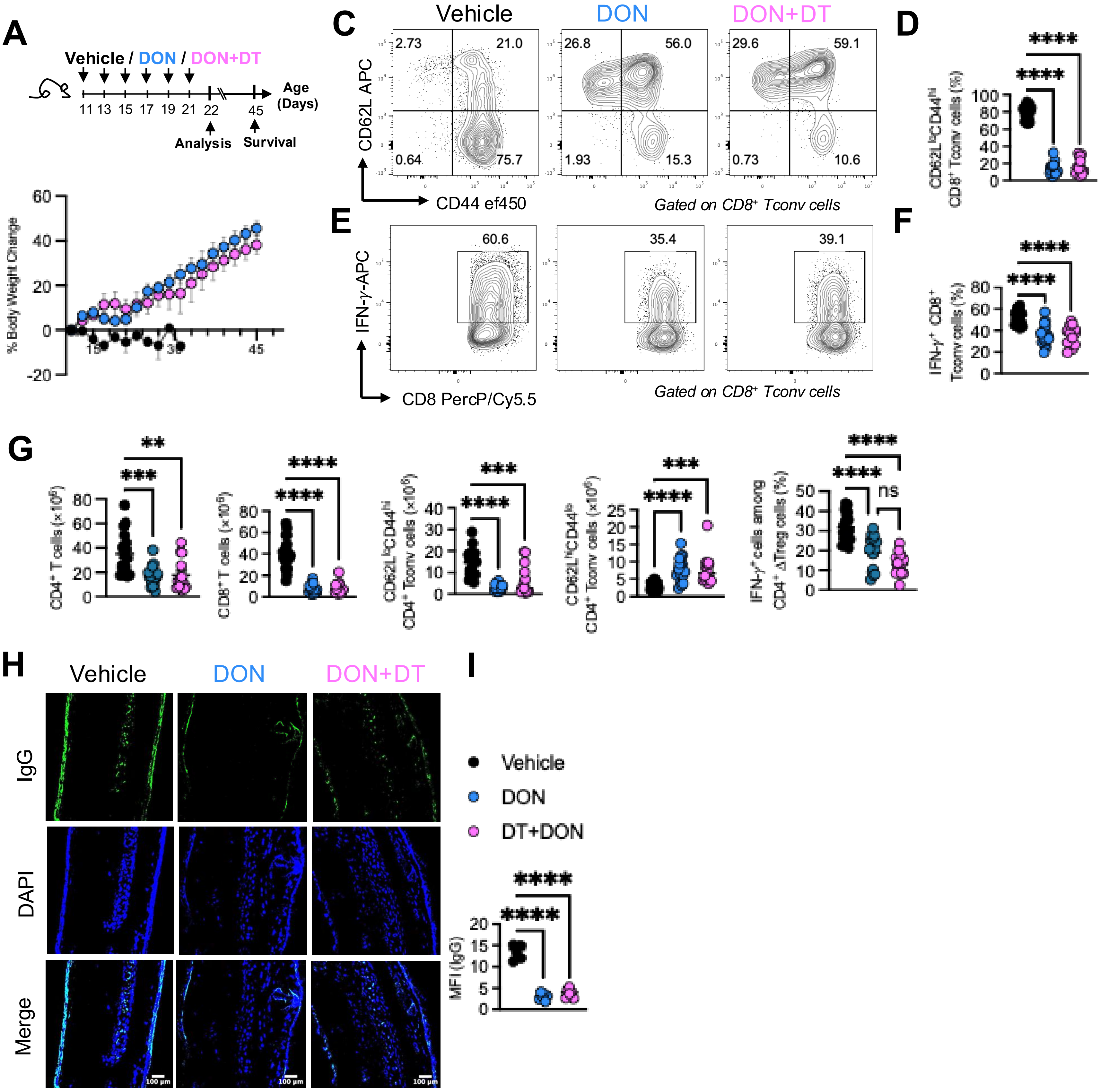

A
Vehicle / DON / DON+DT
11 13 15 17 19 21 22 45
Age (Days)
Analysis
Survival
% Body Weight Change
C
Vehicle
DON
DON+DT
CD62L APC
CD44 ef450
2.73
21.0
0.64
75.7
26.8
56.0
1.93
15.3
29.6
59.1
0.73
10.6
Gated on CD8+ Tconv cells
D
CD62L hiCD44hi CD8+ Tconv cells (%)
E
IFN-γ-APC
CD8 PercP/Cy5.5
60.6
35.4
39.1
Gated on CD8+ Tconv cells
F
IFN-γ+ CD8+ Tconv cells (%)
G
CD4+ T cells (×10^6)
CD8+ T cells (×10^6)
CD62L loCD44hi CD4+ Tconv cells (×10^6)
CD62L hiCD44lo CD4+ Tconv cells (×10^6)
IFN-γ+ cells among CD4+ ΔTreg cells (%)
ns
H
Vehicle
DON
DON+DT
IgG
DAPI
Merge
100 μm
I
Vehicle
DON
DT+DON
MFI (IgG)

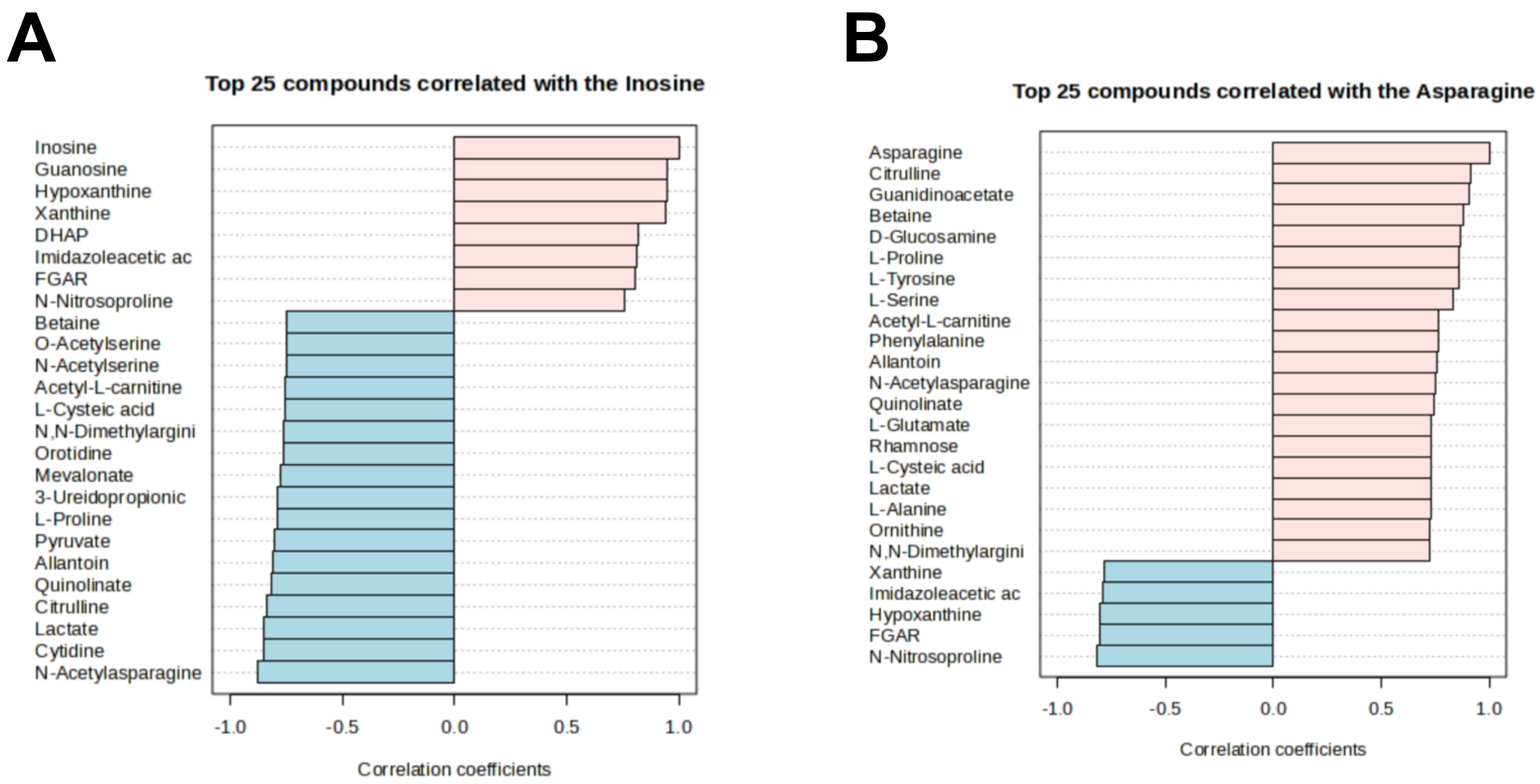
A
Top 25 compounds correlated with the Inosine
Inosine
Guanosine
Hypoxanthine
Xanthine
DHAP
Imidazoleacetic ac
FGAR
N-Nitrosoproline
Betaine
O-Acetylserine
N-Acetylserine
Acetyl-L-carnitine
L-Cysteic acid
N,N-Dimethylargini
Orotidine
Mevalonate
3-Ureidopropionic
L-Proline
Pyruvate
Allantoin
Quinolinate
Citrulline
Lactate
Cytidine
N-Acetylasparagine
-1.0 -0.5 0.0 0.5 1.0
Correlation coefficients
B
Top 25 compounds correlated with the Asparagine
Asparagine
Citrulline
Guanidinoacetate
Betaine
D-Glucosamine
L-Proline
L-Tyrosine
L-Serine
Acetyl-L-carnitine
Phenylalanine
Allantoin
N-Acetylasparagine
Quinolinate
L-Glutamate
Rhamnose
L-Cysteic acid
Lactate
L-Alanine
Ornithine
N,N-Dimethylargini
Xanthine
Imidazoleacetic ac
Hypoxanthine
FGAR
N-Nitrosoproline
-1.0 -0.5 0.0 0.5 1.0
Correlation coefficients

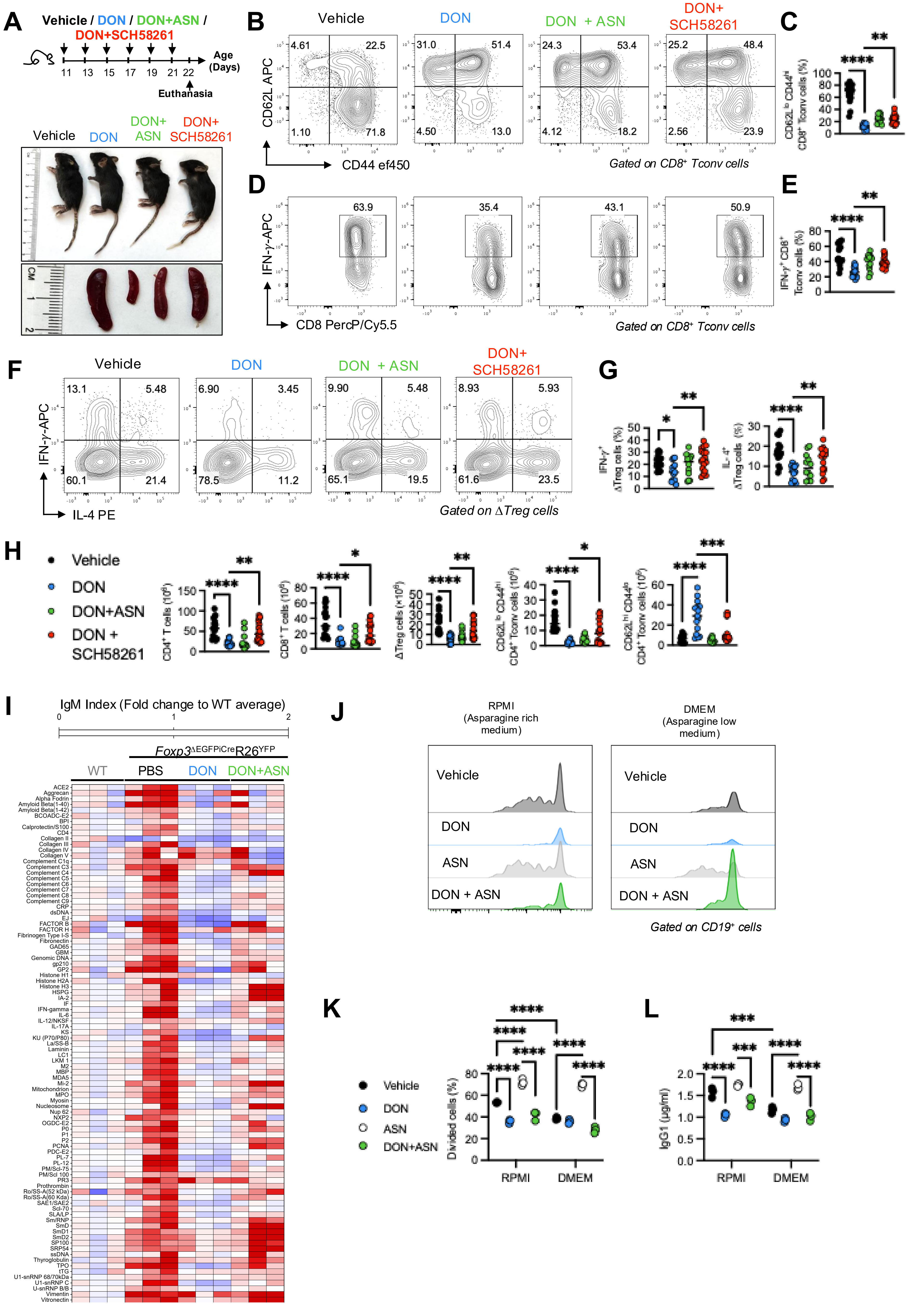
A
Vehicle / DON / DON+ASN / DON+SCH58261
Age (Days)
11 13 15 17 19 21 22
Euthanasia
Vehicle DON DON+ ASN DON+ SCH58261
B
Vehicle DON DON + ASN DON+ SCH58261
CD62L APC
CD44 ef450
Gated on CD8+ Tconv cells
C
D
IFN-γ-APC
CD8 PercP/Cy5.5
Gated on CD8+ Tconv cells
E
F
IFN-γ-APC
IL-4 PE
Gated on ΔTreg cells
G
H
Vehicle
DON
DON+ASN
DON + SCH58261
I
IgM Index (Fold change to WT average)
Foxp3ΔEGFPiCreR26YFP
WT PBS DON DON+ASN
J
RPMI (Asparagine rich medium)
DMEM (Asparagine low medium)
Vehicle
DON
ASN
DON + ASN
Gated on CD19+ cells
K
Vehicle
DON
ASN
DON+ASN
Divided cells (%)
RPMI DMEM
L
IgG1 (μg/ml)
RPMI DMEM
